# Inherent aggressive character of invasive and non-invasive cells dictates the *in vitro* migration pattern of multicellular spheroid

**DOI:** 10.1038/s41598-017-10078-7

**Published:** 2017-09-14

**Authors:** Sukanya Gayan, Abhishek Teli, Tuli Dey

**Affiliations:** Institute of Bioinformatics and Biotechnology,Savitribai Phule Pune University, Pune, India

## Abstract

Cellular migration, a process relevant to metastasis, is mostly studied in the conventional 2D condition. However, cells cultured in the 3D condition assumed to mimic the *in vivo* conditions better. The current study is designed to compare an invasive and non-invasive adenocarcinoma cell with an invasive fibrosarcoma cell to understand the migration pattern of the multicellular spheroid. It is observed that conventional haplotaxis, chemotactic and pseudo-3D migration assay cannot distinguish between the invasive and non-invasive cells conclusively under 2D condition. Invasive spheroids migrate rapidly in sprouting assay in comparison to non-invasive spheroids. Effects of cytochalasin B, marimastat and blebbistatin are tested to determine the influence of different migration modality namely actin polymerization, matrix metalloprotease and acto-myosin in both culture conditions. Altered mRNA profile of cellular migration related genes (FAK, Talin, Paxillin, p130cas and Vinculin) is observed between 2D and 3D condition followed by the changed expression of matrix metallo proteases. A distinct difference is observed in distribution and formation of focal adhesion complex under these culture conditions. This study demonstrates the efficacy of multicellular spheroids in identifying the intrinsic aggressive behavior of different cell lines as a proof of concept and recognizes the potential of spheroids as a migration model.

## Introduction

Metastasis or migration of cancer cells to specific tissues still remains a mystery and a challenge to the scientific world in many aspects. Understanding the molecular and cellular basis of such a complex process will certainly enhance the basic knowledge and thereby facilitate its translation into diagnostic and therapeutic advancements. Traditional 2D assays have provided supporting information that has lead to the understanding of the cellular migration process in terms of the involvement of cytoskeletal structure, focal adhesion complex, hundreds of accessory proteins, kinases, GTP coupling proteins and kinase dependent extracellular receptors. Recent studies have included environmental cues, mechanical force, and other biochemical moieties as significant players in cell-substratum interactions and migration^1^. However, the information obtained from the 2D culture cannot mimic the *in vivo* observations as proved in many instances. This inconsistency led to the rise of the new school of three dimensional (3D) cultures in early twentieth century^2, 3^. Preliminary studies of cellular aggregate and matrix based multicellular structure exhibit the potential of 3D culture in the field of tumor biology and therapeutics in terms of drug resistance^4–6^. It is observed that in 3D culture condition cells have altered gene expression and protein expression profile compared to 2D culture^7, 8^. Cancer cells cultured in 3D condition mimic the oxidative and nutrient gradient as observed in avascular tumors. Different biomaterials have been explored to emulate the native 3D environment such as collagen^9, 10^ and isolated extra cellular matrix like matrigel^11^. Other components like synthetic polymer (e.g. polyethylene glycol), natural protein (e.g. silk fibroin) and carbohydrate (e.g. alginate) are also used to fabricate scaffold and mimic tumor model^12–16^. The popularity of these models lies in their commercial availability, reproducibility and technological ease of use.

The significance of three dimensional culture systems is undeniable as different modes of migration namely; mesenchymal, amoeboidal and hand over hand are identified from the 3D culture only. Such diverse mode of migrations is proven to be crucial for *in vivo* metastasis process^17–19^. Identification of transitional migration (mesenchymal to amoeboidal or epithelial to mesenchymal) represents the potential of the 3D model and its ability to mimic the complexity and adaptability of tumors during therapy^17, 20, 21^. Portrayal of cellular migration within synthetic or natural polymer based scaffolds depends on certain factors such as scaffold chemistry, stiffness, pore size etc which has been found to influence migration speed and mode significantly^22, 23^. Other migration controlling factors such as focal complex, plaque and points are found to be critical for cellular migration in 2D condition, but their exact role in 3D culture is not clear yet^24–27^. Among the reported focal adhesion proteins in 2D assays, very few such as vinculin, p130CAS, and ezrin are found to be critical for 3D invasion and spheroid growth^28, 29^. Exact role of focal adhesion kinase (FAK) in 3D migration is not properly understood, though it plays a significant role in cancer and metastasis^30^. Additionally, acto-myosin contractility, pseudopodia activity and matrix metalloproteinase (MMP) mediated matrix deformation has also been found to play important roles in 3D scaffold migration, irrespective of their negligible role in 2D migration. The impact of the MMPs in tumor is critical, as broad-spectrum MMP inhibitors are hailed as potential drugs^12, 31^. Among the MMPs, secretary ones (MMP2 and MMP 9) and the surface bounds are widely studied and has been found to be strongly associated with aggressive and metastatic breast cancer^32–35^.

As a model system, multicellular spheroids are fundamentally cellular aggregation of diameter 100–500 µm which can establish the pathophysiological gradients within and comparable to the situation in tumor nodules. Spheroids are grown from a small aggregate of cell without any foreign matrix and secrete extracellular matrix to create tissue like microenvironment including hypoxia^36^. Multicellular spheroids are used frequently for high throughput drug screening, therapeutic assay and neoangiogenesis assay^37–40^ but not used successfully as a migration model. In fact, in spite of its acceptance as a tumor model, it was never evaluated and validated as a potential model for migration study until very recently^41^. One possible reason for such omission might be that while most of the cancerous cells grow perfectly within scaffold/hydrogel structure; very few of them are amenable to the multicellular spheroid formation. The unpredictability of the spheroid invasion assays and their lack of uniformity may have further acted as a hindrance for such studies^42^. All these challenges can be comprehended from the fact that only a few reports are available in this regard and that too with varying degree of contradictory data. For example, one report hailed the potential of 3D spheroid as a model of epithelial-mesenchymal transition (EMT) while another stated the morphology and behavior of aggregation of invasive cells were not distinguishable from noninvasive cells^43, 44^.

With this background, the present study is designed to understand the cellular migration pattern of 3D multicellular spheroid using two invasive (HT 1080 and MDA MB 231) and one non-invasive (MCF7) cancerous cell lines^45, 46^. The study is intended to answer the following questions, (i) whether the cells designated as highly invasive and poorly invasive one, perform accordingly within a 2D and 3D environment, (ii) which kind of techniques augment the inherent behaviour of the cells in terms of migration, (iii) which cellular machinery is indispensable for migration and (iv) what is the profile of different molecular participants of cellular migration such as focal adhesion proteins and MMPs in 2D culture and 3D spheroids.

## Methods and Materials

### Materials

DMEM, fetal bovine serum, penicillin/streptomycin, DAPI, Triton × 100, DMSO, Trypsin-EDTA, cytochalasin B and other chemicals used, were of cell biology grade and procured from Himedia, India. Anti Vinculin antibody conjugated with FITC and TRITC-conjugated phalloidin, Spheroid sprouting assay kit (Cultrex^TM^), marimastat and blebbistatin were brought from Sigma, Mt. Louise, USA. cDNA synthesis kit (RevertAid™ First Strand cDNA Synthesis Kit, Fermentas) and PCR kit (GoTaq® Green Master Mix, Promega) were brought from Thermo fisher Inc. Primers for semi Q RT-PCR were procured from Eurofin, India. Trizol, RIPA buffer and Transwell (8 µm pore, PET membrane) were procured from Invitrogen Biosciences. Glass and plastic wares were brought from Milipore and Tarson, India.

### Cell culture

Invasive and non-invasive adenocarcinoma cells lines, such as MDA MB 231, MCF7 respectively and invasive fibrosarcoma cell line HT 1080 were procured from NCCS, Pune, India. Cells were cultured in high glucose (4.5 gm/lt) DMEM medium with 10% FBS and 1% penicillin/streptomycin under standard cell culture conditions at 37 °C in humidified CO_2_ incubator. Cells were grown in T25 flask to reach 70% confluency and harvested further using Trypsin-EDTA (2.5%) solution.

### Fabrication of 3D spheroid

Harvested cells were used to prepare 3D spheroid. Briefly, cell culture treated multiwell plates were coated with 1% agarose solution to prepare a non-adhesive surface. 5000 cells per well were seeded on agarose coated wells and the multi well plates were then centrifuged at 2000 RPM for 2 min to bring the cells together through centrifugal force. Cellular micro-complexes were further incubated at standard cell culture condition to facilitate the spheroid formation over next 3–5 days.

To fabricate single spheroid, 1× spheroid formation solution (Cultrex^TM^, Sigma) was added to 96well round bottom cell culture treated plate followed by the addition of 3000 cell/wells. The cells in the well plates were further centrifuged at 200RCF for 2 min and incubated in standard cell culture conditions for 3 days.

### Characterization of 3D spheroid

Spheroids on multiwell plate were monitored and imaged *in situ* with phase contrast mode of Nikon Eclipse Ti-U over time. For scanning electron microscopy (Jeol JSM 630/OA), single spheroids were collected using wide mouth tips and fixed with 2% paraformaldehyde (for 1 hr) followed by serial dehydration with 70–100% ethanol. Samples were further placed on glass cover slip and subjected to platinum sputtering before imaging. Imaging was done at 20 kV at 1000–1500× magnification.

### Cellular migration and inhibition in 2D platform

Cellular migration of individual cell lines under 2D condition was investigated by wound healing assay^47^. Briefly, multi well plates were seeded with 10,000 cells and incubated for 24–48 hr. Confluent cell layers were further scratched with sterile tip (10 µl). Surrounding cells were allowed to fill the scratch under standard conditions and imaging was done at different time points (0, 3, 6 and 24 hr) using phase contrast microscope (Nikon Eclipse Ti-U) to calculate the cellular migration rate. Wound distance was measured by NIS-Elements software and migration rate was calculated by the following formula and plotted as mean ± S.E.M (standard error of the mean).

Migration rate = (Distance of the wound at 0 hr -Distance of the wound at 6 hr)/Elapsed time (6 hr).

Inhibitors such as Cytochalasin B^48^, Blebbistatin^49^ and Marimastat^50^ were added (10 µM) to the wound and wound healing was monitored over time (0, 3, 6 and 24 hr). Inhibition efficiency was calculated as percentage inhibition of wound healing using the following formula and plotted as mean ± S.E.M.

Percent inhibition of wound healing = (Final distance of the wound at 24 hr/Initial distance of the wound at 0 hr) × 100.

### Migration of cells and spheroids on transwell

Transwell inserts were equilibrated with complete medium beforehand for 24 hr. Cells from 2D culture (10,000/well) were seeded into the insert with serum deprived medium. The lower chamber were filled with 600 µl of complete media (with 10% FBS) to function as chemotactic agent. The transwell were incubated under standard cell culture conditions for 24–48 hr. Thereafter the bottom side of the transwell inserts was incubated with MTT (0.5 mg/ml) solution for 4 hr to create formazan crystals within migrated live cells. Formazan crystals were dissolved in DMSO and optical density was measured at 595 nm. Optical density was plotted as mean value ± S.E.M.

### Pseudo3D cellular migration of Spheroid on 2D surface

3D spheroids of individual cell lines were collected from the multi well plate using wide mouth tips and deposited on sterile cover slips^41^. Cover slips were further incubated under cell culture conditions without disturbing the spheroids for 12–24 hr. Phase contrast image (Nikon Eclipse Ti-U) of the dissolved spheroids were captured at different time points (0, 2, 4, 24 hr). Pseudo 3D migration (reversal) rate was calculated by measuring the initial and final coverage area (NIS-Elements software) and normalizing it. Ratio of Final/initial area was plotted as mean value ± S.E.M.

### Spheroid migration in 3D sprouting assay and inhibition of sprouting

3D spheroids were fabricated as per the manufacture’s protocol (Cultrex^TM^ Spheroid formation assay) and single spheroid was subjected to spheroid sprouting assay (Cultrex^TM^ Sprouting assay) for the time period (24–72 hr) as recommended in the manufacturer’s protocol. Briefly, the spheroid was overlaid with complete DMEM medium and chemotactic agent (laminin, 1 µg/µl) and spheroid invasion reagent. The sprouting of spheroids were imaged over time (after 6^th^ day) using phase contrast microscope (Nikon Eclipse Ti-U).

Migration inhibitors, cytochalasin B, blebbistatin and marimastat were added as stated before to the spheroid invasion matrix along with laminin. Further imaging was done on 6^th^ day to analyze the inhibition.

### Zymogram analysis for MMP secretion

Gelatin zymogram assay was used to analyze the secretory and membrane bound MMP level of 3D spheroids and cells isolated from 2D culture. Cells and spheroids were collected and lysed using RIPA buffer. Protein content in spent media, cell lysate and cell pellet was quantified and used for gelatin zymogram. As described elsewhere, gelatin (0.1%) containing 10% SDS-PAGE gels were run under standard conditions of Lamellie with 1 µg of total protein. Further the gels were treated with reconstitution buffer and activated with activation buffer for 12 hr. Collagenase was used as a positive control. Different MMP bands were visualized under white light and analyzed further.

### mRNA profiling of migration specific genes

Individual cell lines grown as 2D and 3D spheroids were harvested and subject to total RNA isolation using Trizol solution. Isolated RNA was quantified and used for cDNA synthesis (Fermentas RevertAid First Strand cDNA Synthesis Kit) following the manufacturer’s protocol. Semi quantitative RT PCR analysis of five migration specific genes (FAK, p130CAS, Vinculin, Paxilline and Talin) was done for 20 cycles following the manufacturer’s protocol (GoTaq Promega). Beta-2-microglobulin (B2M) was used as an internal control^51^. Densitometric profiling of amplicon band intensity was done by ImageJ software^52^. Band intensity of each gene was normalized with B2M and plotted as mean ± S.E.M.

### Focal adhesion complex and actin distribution in 2D and 3D spheroid condition

Cells cultured on 2D surface and spheroids were subjected to vinculin, actin cytoskeleton and nucleus staining to analyze the distribution of focal adhesion complexes and cellular cytoskeleton. Briefly the adhered cells and spheroids were fixed with 2% paraformaldehyde and permeabilized with 0.01% Triton × 100. Anti Vinculin monoclonal antibody conjugated with FITC (green) and TRITC-conjugated phalloidin (red) were used in 1:150 and 1:250 dilutions respectively. Nucleus staining was done with DAPI (blue) (1:5000 dilutions). Imaging was done using Nikon AR1 confocal microscope using the Z scanning mode. Images were analyzed using NIS Elements software. Distribution of vinculin throughout the cells was quantified by total fluorescence measurement using ImageJ software. Measured values were plotted and compared between 2D and 3D condition of each cell lines.

### Statistical analysis

All the experiments were repeated three times with technical triplicates. Plotted values were mean ± S.E.M. Difference within the groups was analyzed by one way ANOVA followed by post hoc analysis (Tukey’s test) to compare the significant level of differences between each group. Comparative analysis of mRNA level between the 2D and 3D samples of individual cell lines were done by Student’s T test. In all cases significance level was assumed 0.05 and p value < 0.05 considered significant.

## Results

### Spheroid fabrication and characterization

**Figure 1:**
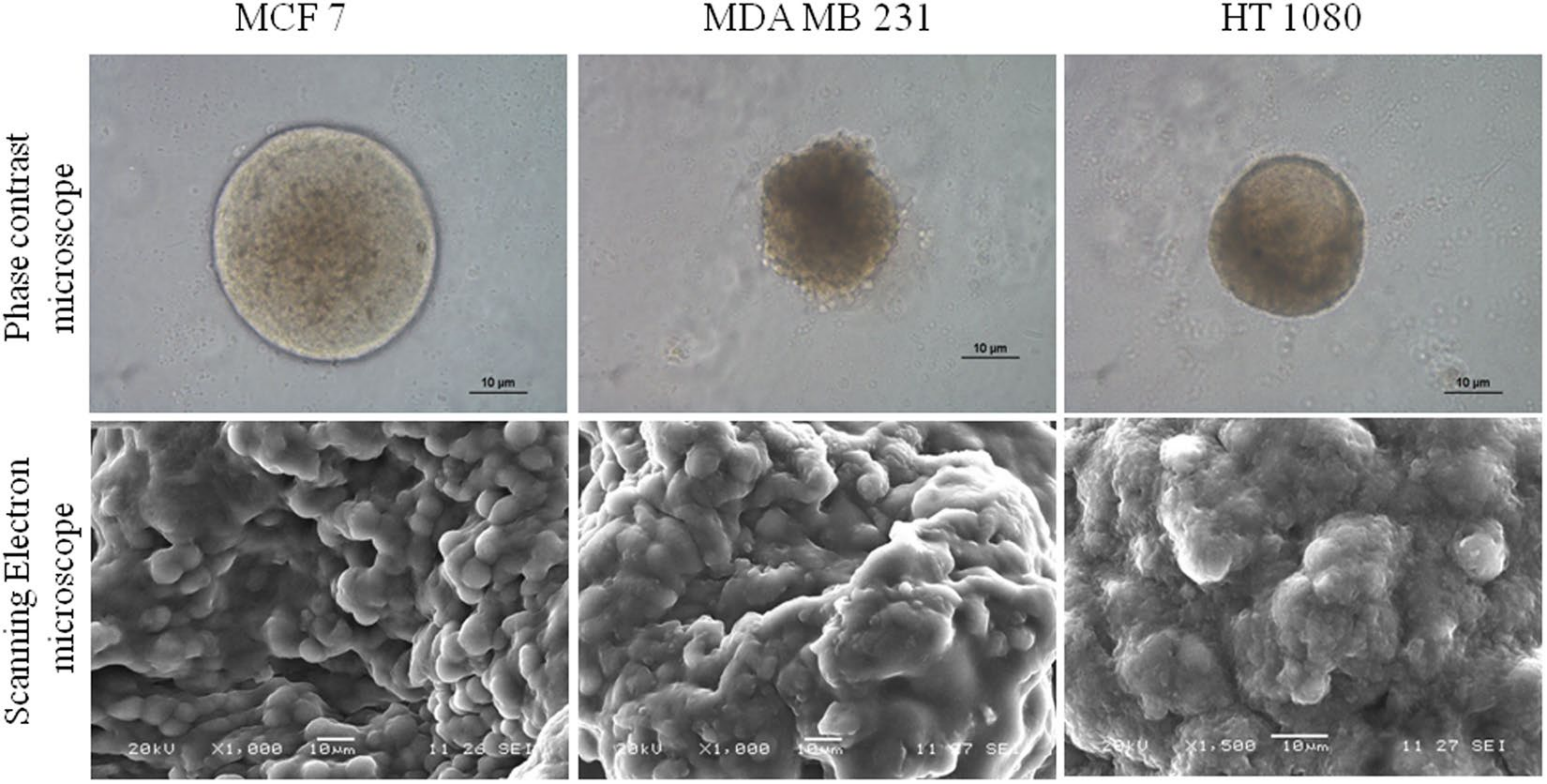
Microscopic images of the spheroid. Phase contrast and scanning electron microscopic images of MCF7, MDA MB 231 and HT 1080 spheroids grown in spheroid invasion assay matrix. Scale bar in both panels is 10 µm.

As observed from the Fig. 1, multicellular spheroids of 100–150 µm diameters are formed with three cell lines. Morphology of spheroids prepared with spheroid formation matrix (Cultrex™, Sigma) is found to be more compact and dense in nature, while spheroids formed in agar matrix show relatively less aggregation (SI Fig. 1). From phase contrast microscopy, both MDA MB 231 and HT1080 spheroids appear to be smaller than the MCF7 ones. It’s also clear that MCF7 spheroids exhibit a clear boundary around the spherical structure, which is absent in both MDA MB 231 and HT1080 spheroids. Scanning electron micrographs reveal fused and conglomerated appearance in all three spheroids which indicate intercellular connection.

### Wound healing assay in 2D condition and migration inhibition

**Figure 2:**
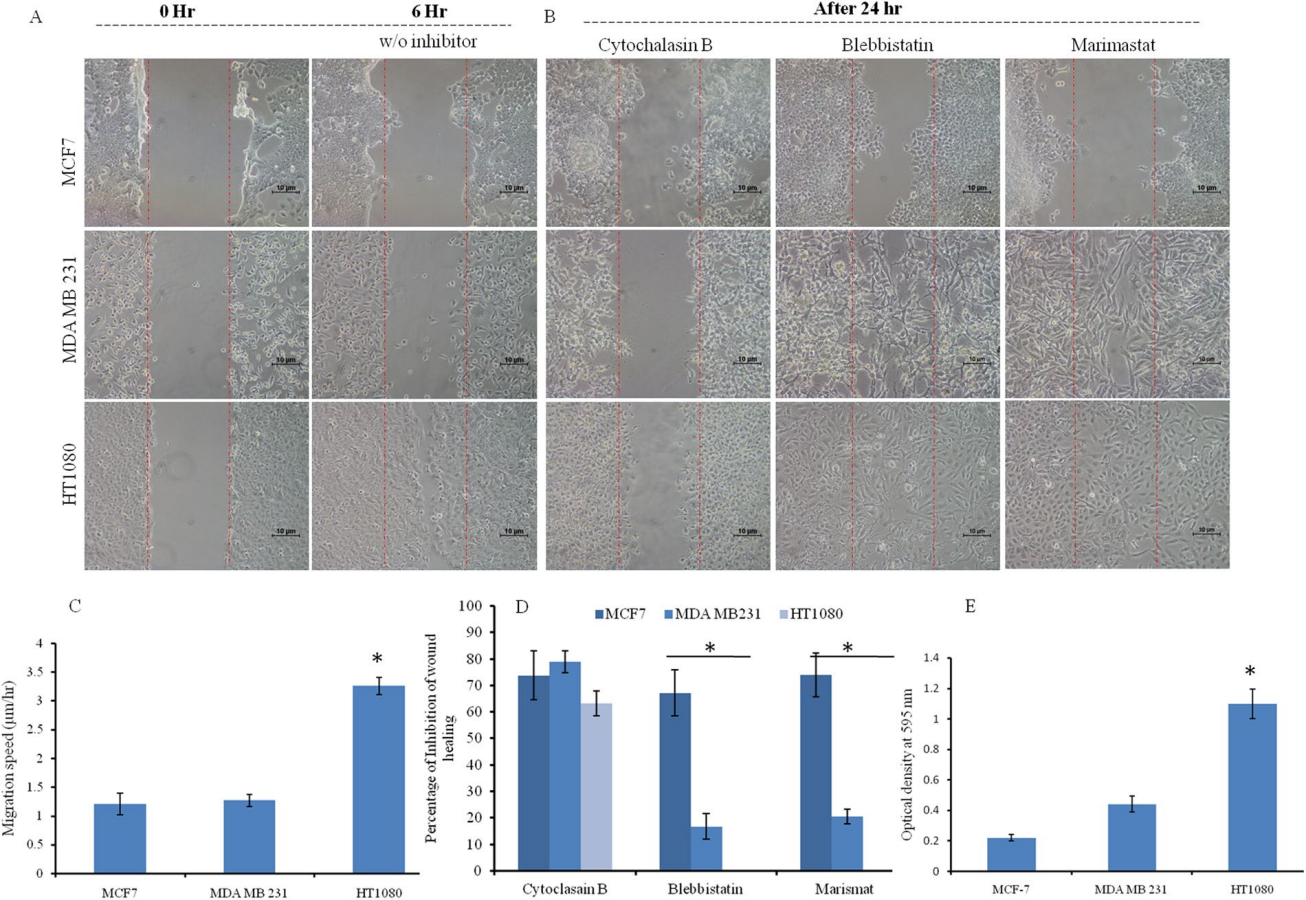
Cellular migrations and inhibition analysis in the 2D platform of MCF7, MDA MB 231 and HT 1080 cells. (**A**) Confluent cultures are subjected to wound formation and subsequent healing over 0–6 hr. Imaging is done with phase contrast mode of Nikon Eclipse Ti-U. The scale bar is 10 µm. (**B**) Migration inhibitor’s (Cytochalasin, Marimastat, and Blebbistatin) effect on wound healing is analyzed over 24 hr and imaging is done as before. (**C**) Cellular migration rate on the 2D surface is calculated and plotted (mean ± SEM). HT 1080 is identified as the most aggressive in terms of migration. (**D**) Percent inhibition of wound healing is calculated and plotted (mean ± SEM). The efficiency of inhibitors on different cell lines is analyzed statistically. (**E**) Transwell migration assay of MCF7, MDA MB 231 and HT 1080 cells is done. Chemo-tactically migrated cells are quantified and plotted (mean ± SEM). All data are analyzed by one-way ANOVA and post hoc Tukey’s test. (* denotes p < 0.05 in every case).

Wound healing (haplotaxis) and inhibition of migration within 0–24 hr is described in Fig. 2. Cells are allowed to fill the wounds of same dimension and it is observed that HT1080, being a fibrosarcoma cell line, covers the wound within 6 hr, while MCF7 and MDA MB 231 cells fill the wound by 24 hr (Fig. 2A and SI Fig. 2). Rate of migration among the three cell line is calculated and compared to find that HT1080 is the fastest migrating cell among the three. MDA MB 231 and MCF 7 cells are found to have comparatively similar speed on 2D surface (Fig. 2C).

Cytochalasin B, marimastat and blebbistatin are used to analyze their inhibitory effects on 2D migration over 24 hr after creating the wound (Fig. 2B). Inhibition of wound healing is maximum in MCF7 cell line for all three inhibitors (Fig. 2D). Statistically, all three cell lines show no significant difference between each other for cytochalasin B mediated inhibition (~70% inhibition). However, marimastat and blebbistatin are found to have zero or negligible effect on migration of both HT1080 and MDA MB 231 cells respectively. For blebbistatin, HT1080 and MDA MB 231 both differ significantly from MCF 7 but not from each other, while for Marimastat all three of them differ from each other significantly (p < 0.05).

### Migration of 2D cell population through transwell

Chemotactic migration rate is measured by the transwell assay using fetal bovine serum as chemotactic agent. Cellular migration to the bottom part of the transwell inserts are quantified by the formazan crystals which is directly proportionate to the migrated cell number. As observed from the Fig. 2E, chemotactic migration towards complete cell culture medium (10% FBS) is fastest in HT1080 cells followed by MDA MB 231. MCF7 exhibits slowest chemotVactic migration rate. Interestingly, HT 1080 differs significantly from the other two cell lines but those two (MDA MB 231 and MCF7) do not differ significantly from each other.

### Pseudo 3D migration on 2D surface

**Figure 3:**
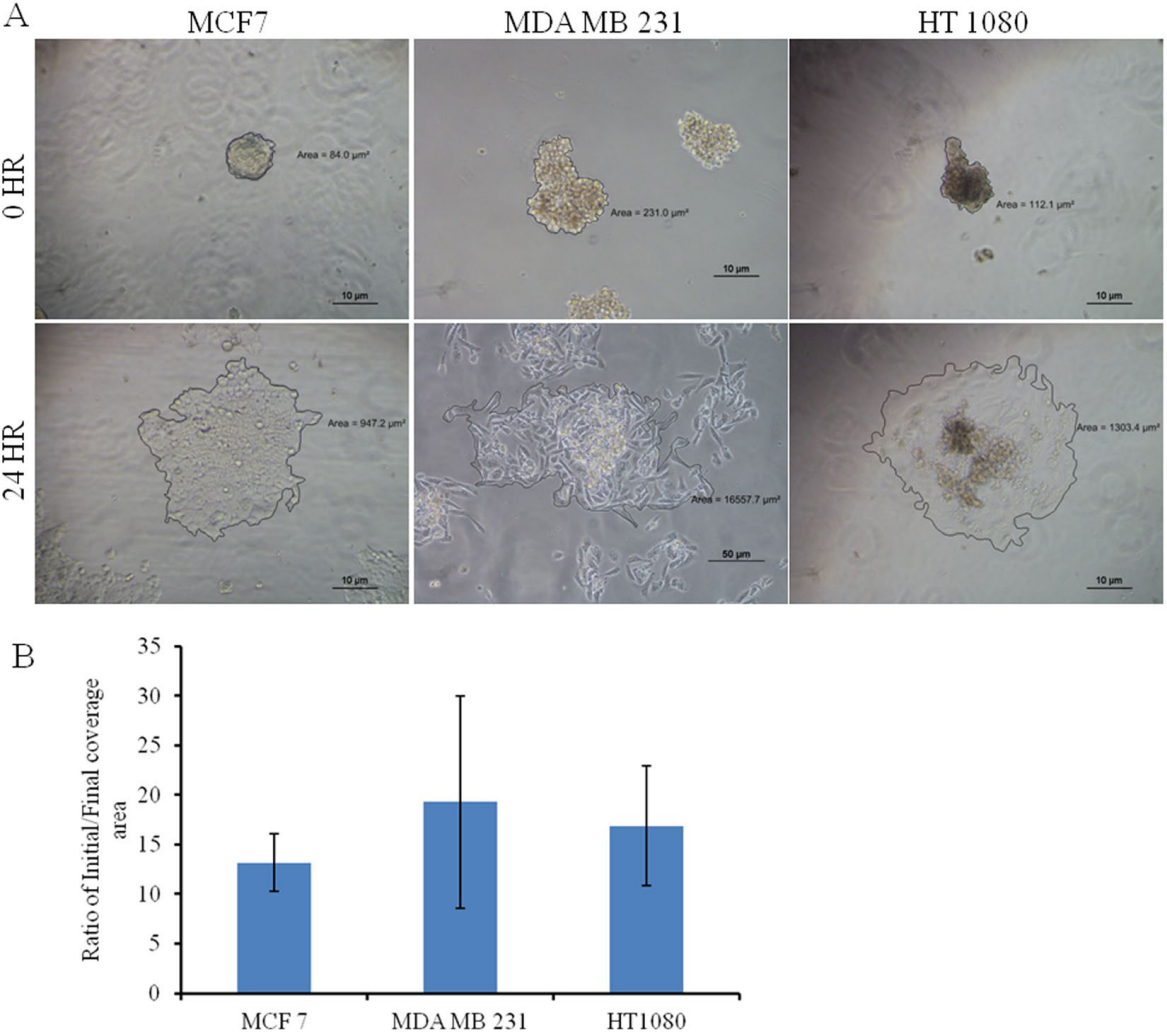
Pseudo 3D migration of MCF7, MDA MB 231 and HT 1080 spheroid on the 2D surface. (**A**) A single spheroid is incubated on the glass coverslip and incubated for 24 hr. Reversal /melting of spheroids over time is analyzed by imaging with phase contrast mode of Nikon Eclipse Ti-U. The scale bar is 10 µm. (**B**) The covered area is calculated (NIS Elements software) and normalized with initial spheroid size for plotting (mean ± SEM).

Single spheroids of different cell lines are placed on top of the cover slip and monitored to dissociate over time (24 hr) (SI Fig. 3). Figure 3A exhibits the rate of complete spreading of MCF7, HT1080 and MDA MB 231 spheroid. The spheroid melting or reversal of spheroids primarily depends on the spheroid size and invasive nature of the cells. As observed from Fig. 3B, MCF7 and HT1080 exhibit comparatively similar coverage ratio while MDA MB 231 shows highest coverage area (statistically not significant).

### Spheroid sprouting assay without and with inhibitors

**Figure 4:**
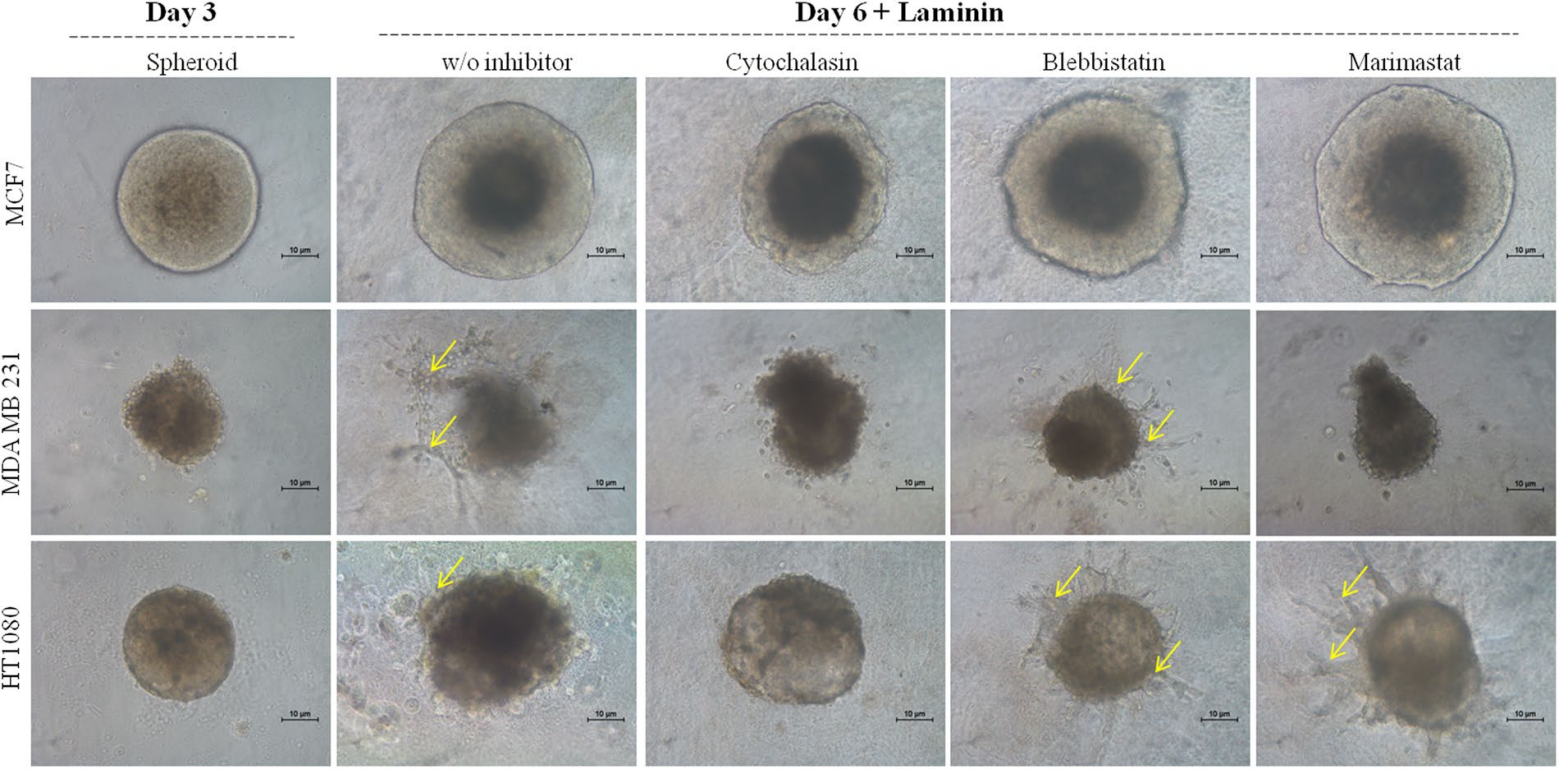
Spheroid sprouting assay of MCF7, MDA MB 231 and HT 1080 cells with and without inhibitors. Single spheroids grown in spheroid formation assay (CultrexTM) are subjected to the chemo-attractant (laminin) dependent migration in absence and presence of inhibitors over time. Imaging is done with phase contrast mode of Nikon Eclipse Ti-U. The scale bar is 10 µm. Yellow arrows indicate sprouts.

Spheroid migration is further analyzed by sprout formation assay using single spheroid and laminin as chemotactic agent. As observed from Fig. 4, MDA MB 231 and HT 1080 spheroid initiate sprout formation, after 72 hr while MCF 7 does not exhibit any sprout formation. This result supports the invasive and highly invasive nature of MDA MB 231 and HT 1080 cells and non-invasive behaviour of MCF7 cells as observed in previous literature^44, 45^.

However, incubation with Cytochalasin B, blebbistatin and marimastat show varied level of inhibition of sprouting in invasive cell lines. Treated MCF7 spheroids display no sign of sprouting, but increase considerably in size along with the control (no inhibitor). Both MDA MD 231 and HT1080 show no sprouting in presence of cytochalasin B. Blebbistatin exhibits no visible effect on sprouting in both invasive cells lines. Marimastat Inhibits MDA MB 231 sprouting but has no effect on HT 1080 spheroids.

### Zymogram of cells of 2D culture and 3D spheroid

**Figure 5:**
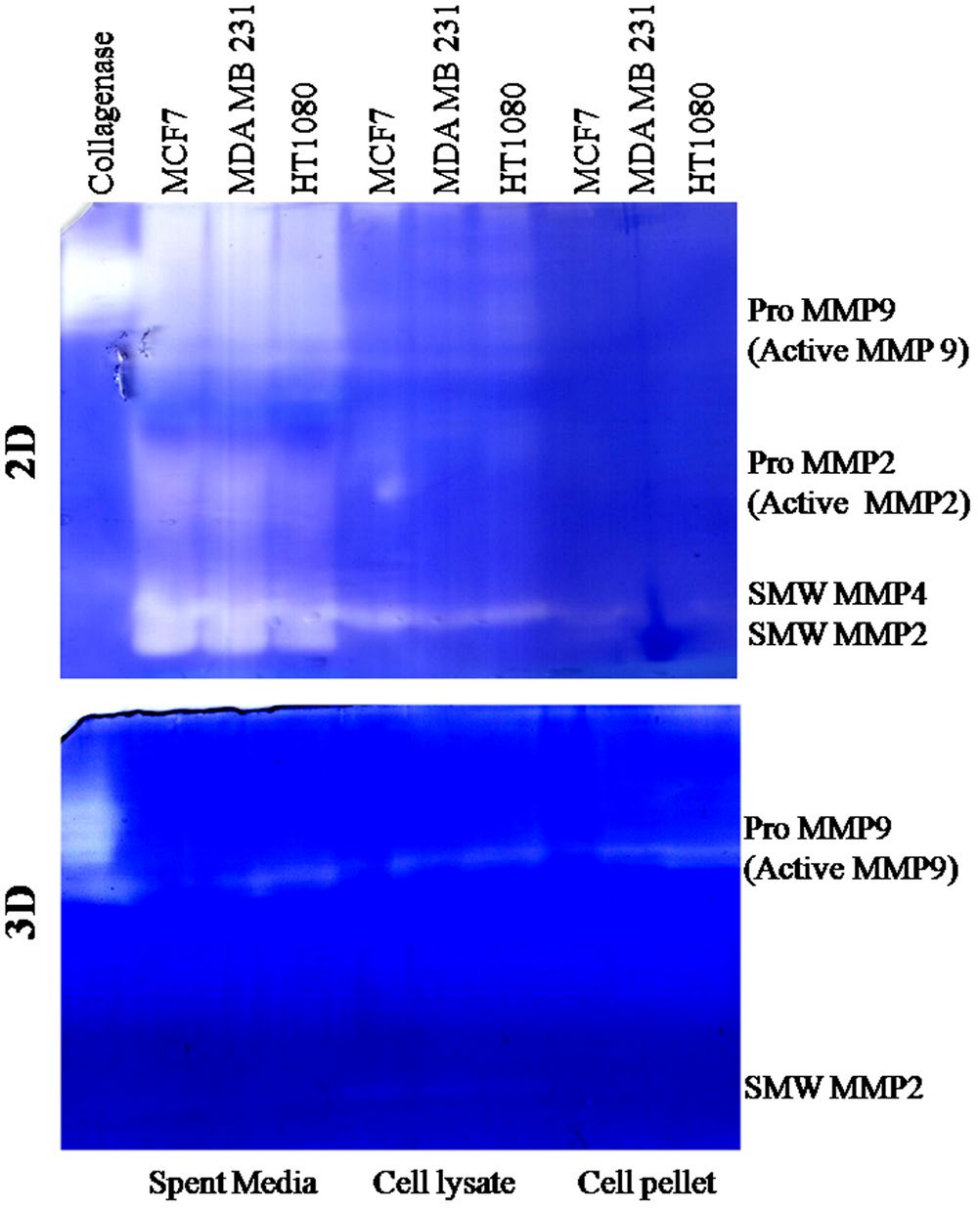
Profile of MMP production in 2D and 3D condition in three cell lines. Zymogram analysis is done using spent medium, cell lysate and cell pellet of two conditions (2D and 3D) of three cell lines. Collagenase is used as positive control. Bands were identified by mol wt.

Zymogram is done using spent medium, cell lysate and cell pellet to analyze the profile of available MMPs, both secreted and membrane bound types (gelatinolytic family). As seen in Fig. 5, all three cell lines cultured in 2D condition secrete four types of MMPs (such as MMP 2, 9, small molecular weight 2 and 4) mostly in the spent medium; smw MMP4 in cytoplasmic fraction and only membrane bound smw MMP2/4 in cell pellet. However in 3D spheroid, all three cell types display decreased amount of MMPs. Pro MMP 9 is present in the spent media, cell lysate and cell pellet of only invasive spheroids (HT 1080 and MDA MB 231). MCF 7 spheroids does not secrete any pro MMP9. Interestingly all types of spheroids exhibit smw MMP 2 in cytoplasmic fraction.

### mRNA profiling of focal adhesion specific proteins

**Figure 6:**
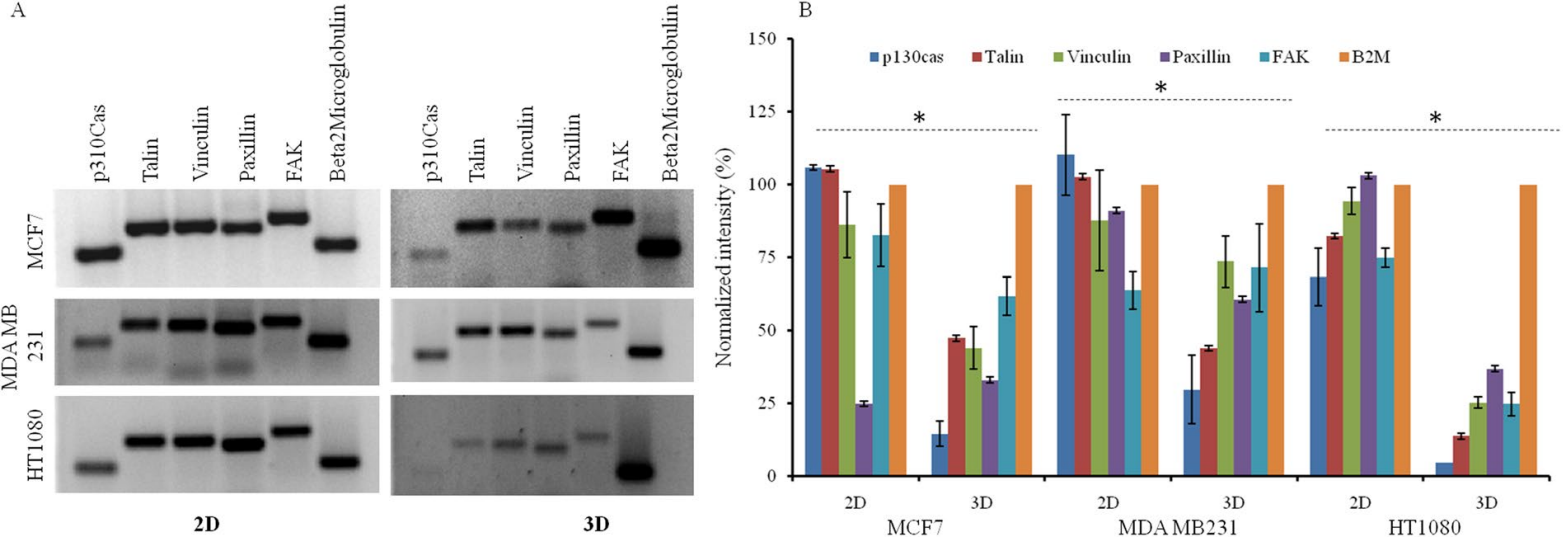
mRNA profiling of migration specific genes in 2D and 3D spheroid condition. (**A**) Representative image of semi-quantitative RT-PCR products of five migration specific genes (p130 Cas, Talin, Vinculin, Paxillin, Focal adhesion kinase) and β2 microglobulin on the agarose gel. (**B**) Densitometric analysis of PCR bands is done using ImageJ software. Statistical analysis (Student’s T test) is done and significant difference between groups (2D and 3D) is identified (* denotes p < 0.05).

mRNA profiles of five focal adhesion specific proteins (Vinculin, Talin, Paxillin, p130cas and FAK) are analyzed in both 2D and 3D spheroid conditions (Fig. 6A) and normalized intensity is plotted (Fig. 6B). As seen all mRNAs are detected in MCF7, HT1080 and MDA MB 231 cells under both 2D and 3D conditions. However the level of occurrence in terms of band intensity is decreased in 3D spheroid in all three cell lines. Decrease in mRNA level is statistically significant among all cell lines between the two conditions (p < 0.05).

### Focal adhesion in 2D culture and 3D spheroid

**Figure 7:**
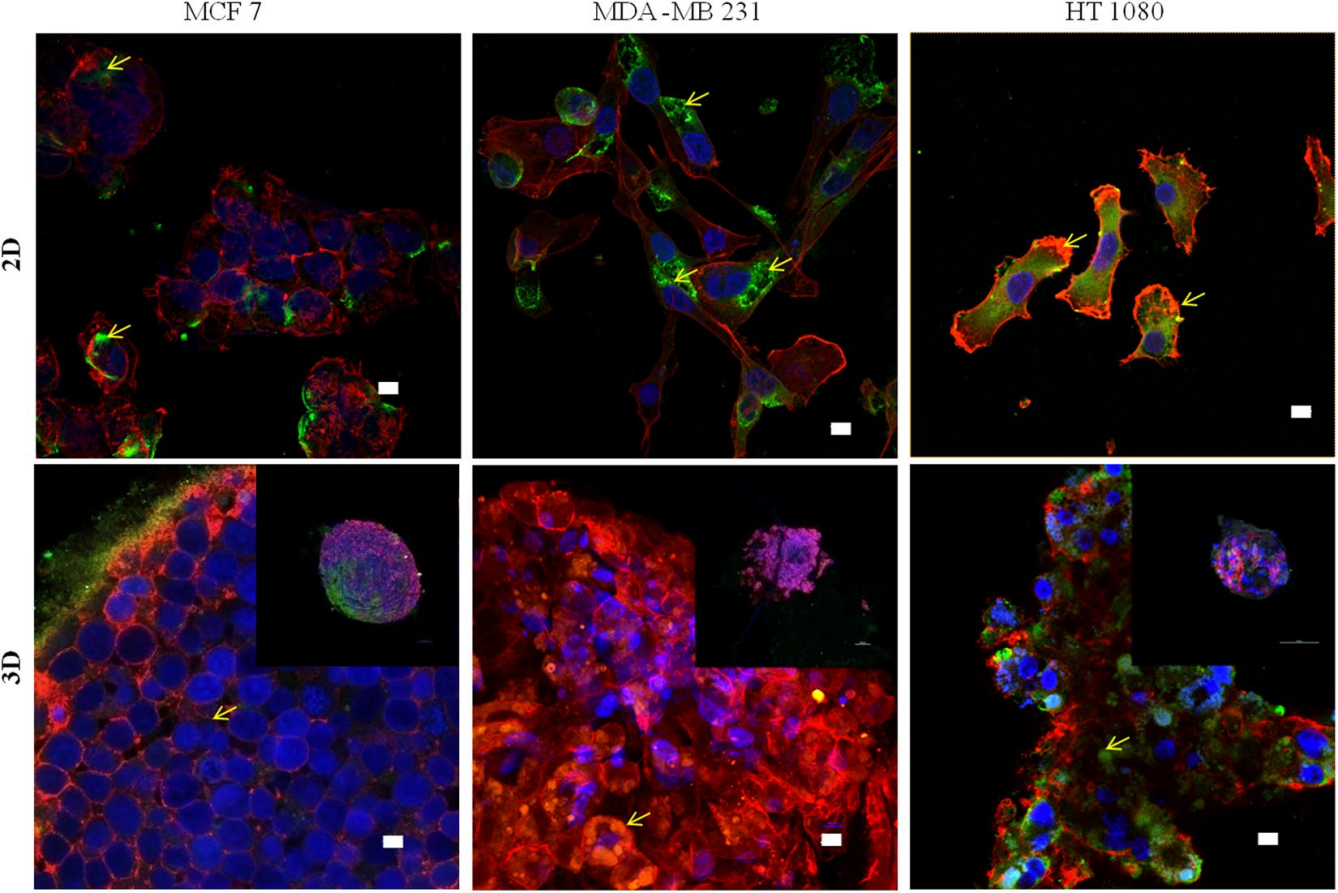
Distribution of actin cytoskeleton and focal adhesion complex protein (vinculin) in 2D and 3D spheroid condition. Cells are grown in 2D (coverslip) and 3D (spheroid) conditions are fixed with 2% PFA and permeabilized with 0.01% Triton × 100. Staining is done with TRITC (red) conjugated phalloidin (actin); FITC (green) conjugated monoclonal anti-vinculin antibody (vinculin) and DAPI (blue) (nucleus). Imaging is done with 405, 488 and 653 nm Laser in Nikon AR1 confocal microscope using 60× oil objective. Spheroid imaging is done with optical Z scanning. The scale bar is 10 µm. Inset images in the lower panel are of a single spheroid in the 10× objective. Yellow arrows indicate vinculin distribution.

Distribution of actin filament and focal adhesion complex formation is investigated in both 2D and 3D spheroid conditions. As seen in Fig. 7, MCF 7 cells in 2D condition shows cortical actin ring, but not filamentous actin network as observed in MDA MB 231 and HT 1080 cells. Distinct focal adhesion point is not detectable in MCF 7 cells, but cytoplasmic vinculin is observed. In both MDA MB 231 and HT 1080, focal adhesion complexes are observed in 2D culture. In spheroid condition, MCF7 shows no distinct focal adhesion complex but minimal cytoplasmic vinculin with ring like actin structure joining the neighboring cells. Interestingly both HT1080 and MDA MB 231 spheroids exhibit cytoplasmic vinculin along with the cortical ring of actin filaments surrounding the cells. Absence of regular network of actin filament is prominent in all of the spheroid structure. Comparative analysis of green fluorescence (vinculin) is done between 2D and 3D conditions using ImageJ which shows decreased vinculin in MCF7 spheroids, equal in MDA MB231 and increased in HT1080 spheroids (SI Fig. 4).

## Discussion

Migration of cancerous cells depends on many environmental factors, as well as on cellular components. Different polymeric scaffolds are used to study cellular migration where the chemistry, stiffness, and porosity of synthetic or natural polymer based scaffolds are found to influence cellular migration mode and speed^12, 22, 23, 53^. Multicellular spheroids have been used as drug screening platform since a long time, due to their ability to mimic the solid tumor organization^36–40^. Though in some recent reports, spheroids are suggested as a model to study metastasis and epithelial to mesenchymal transition, their potential as a platform for cellular migration study has not been evaluated in detail^41–43^.

This study is aimed to evaluate the potential of 3D multicellular spheroid as a migration model. Spheroids are fabricated from two adenocarcinoma and one fibrosarcoma cell line. As observed by the phase contrast, scanning electron microscopic and confocal laser microscopic images, all spheroids indeed show inter-cellular interaction. The phase contrast images clearly differentiate between the invasive cell (MDA MB 231 and HT 1080) spheroids as a loosely bound organization and non-invasive one (MCF7) as more compact structure. However, when observed under scanning electron microscopy, MDA MB 231 and HT 1080 spheroid show diffused cellular entity, while cellular individuality seems to be more intact in MCF7 spheroid.

Cellular migration rate is quantified and compared in the 2D environment by haplotaxis assay (wound healing), and as expected, HT1080 cells of fibrosarcoma origin happen to be the fastest among all three, as they cover the wound within 6 hr. Among the epithelial cells, MCF7 covered the wound completely by 24 hrs followed closely by the MDA MB 231. Though MCF7 is categorized as poorly invasive, its migration in the 2D surface is found to be comparable to the highly invasive MDA MB 231 in the current study. Interestingly, chemotactic migration method distinguishes HT 1080 from the other two. To understand the invasive migration of the spheroids on 2D platform, spheroid reversal assay^41^ (pseudo 3D migration) is carried out. As observed from Fig. 3, disassociation/melting of all three types of spheroid and migration completed within 24 hrs. Normalized coverage of all three spheroids is comparable while MDA MB 231 spheroid fills maximum area. The compact structure and intercellular interaction of the spheroid may influence the disassociation, followed by the migration rate in 2D. It can be assumed that the loss of cellular entity in HT 1080 spheroid may play an important role in delayed reversal of spheroids and thus control the migration rate. It can be assumed that haplotaxis, chemotactic and spheroid reversal assays are not suitable to detect or quantify the invasiveness between the adenocarcinoma cell lines.

Sprouting of spheroids is clearly found to discriminate between the invasive and non-invasive cell types where the invasive spheroids of different origin (HT 1080 and MDA MB 231) exhibit sprouting in presence of chemotactic agent (laminin). The absence of sprouts in MCF7 spheroids can be explained by its non-invasiveness and lack of chemo-tactically inducible population^53^.

Identification of migration controlling factors in both 2D and 3D conditions is done by using three broad spectrum inhibitors^48–50^. Complete inhibition of migration in both 2D and spheroids by cytochalasin B denotes the importance of actin and its polymerization, irrespective of cell types or invasiveness or migration platform. MCF7 cells get inhibited by all three inhibitors in 2D conditions which support its non-aggressive nature. MDA MB 231 and HT1080 shows minimal to no inhibition for both blebbistatin and marimastat respectively. Such resistance towards inhibitors can be stemmed from their aggressiveness and the ability to bypass MMPs/acto-myosin mediated pathways completely^18, 54^. In 3D condition MDA MB 231 sprouting gets inhibited by marimastat while HT1080 spheroids demonstrate no effect of blebbistatin and marimastat. Such variation can be explained by the facts that HT1080 is a more aggressive cell line and being of different origin, it might be less dependent on MMP supported migration than the epithelial ones^55^. Ineffectiveness of blebbistatin in sprouting formation may emphasize the minimal role of acto-myosin contractility in spheroid migration. Further appraisal of marimastat can be done in light of MMPs production, where cells cultured in 2D condition produce all three types (secretory, cytoplasmic and membrane bound) of MMPs and get inhibited by marimastat. The complete absence of MMP9 in MCF7 spheroid further supports its noninvasive nature in comparison to other two invasive spheroids. It can be anticipated that in epithelial cells, secretory MMPs plays an important role in both 2D and 3D migration (Figs 2 and 4) as their inhibition cause no migration in 2D (MCF7) and no sprouting in 3D (MDA MB 231). However, minimal inhibition of MDA MB 231 in 2D and maximum inhibition in 3D can be explained by the fact that invasive breast epithelial cells highly depend on secretory MMPs for their migration in 3D system in comparison to 2D condition^56^.

Migration specific proteins such as p130Cas, vinculin, talin etc play an important role in cell-substratum binding and are also involved in mechanotransduction mediated response^28, 29^. Paxillin and focal adhesion kinase (FAK) play a critical role in integrin mediated adhesion and filopodia formation^30^. As observed, all three cell lines express all five mRNAs in both 2D and 3D condition. However, the level of mRNA decreases significantly in the 3D condition in all three cell lines, which might signify the reduced importance of these proteins in 3D condition. The consequences of this information can be understood better by further probing. Additional analysis of actin cytoskeleton structure and focal adhesion complex distribution are done to understand their role in spheroids. The distribution of actin filaments changed drastically from the 2D to 3D spheroids and is in line with earlier reports^57^. As seen in 2D culture, MCF7 cell has no distinguished filamentous actin, but cortical actin ring structure which is further strengthened in 3D spheroids. The filamentous form of actin cytoskeleton of 2D is converted into the ring like arrangement in 3D spheroids for both MDA MB 231 and HT1080 cells. In MCF7, cytoplasmic vinculin is observed in 2D and get reduced to a point of sparsely detectable signal in spheroids (SI Fig. 4). In both MDA MB 231 and HT 1080, cytoplasmic and focal complex vinculin is observed in the cells grown in 2D culture. In 3D spheroids of both cell types, no detectable structures such as focal point or plaques are observed other than patches of cytoplasmic vinculin. In HT1080 cytoplasmic vinculin patch increases in 3D compared to MDA MB 231 which needs more investigation. Vinculin and its role in mechanotransduction can also be relevant in spheroid environment to sense the spheroid integrity. Previous studies on 3D migration mostly depend on matrix based arrays which identify cell–matrix adhesions, RhoA signaling and actomyosin contractility as universal mechanism^58^. It also speculated that matrix rigidity can be recognized by the acto-myosin dependent mechano-sensors which further control the migration modality^58^. The current report identifies the actin polymerization and protease secretion as the primary driving force of invasive spheroid migration. Reduced acto-myosin contractility and cell-matrix adhesion can be explained by the complete absence of rigid foreign extracellular matrix. As multicellular spheroid organization represents cell-cell interaction more strongly rather than conventional cell-matrix interaction, further questioning of intercellular adhesion and mechano-sensor pathways in spheroids need to be done in molecular level.

## Conclusion

The present study is designed to evaluate and understand the potential of 3D spheroids as a model for cellular migration along with the *in vitro* distinction of invasive and noninvasive cells. It is observed that in 2D condition, invasive breast cancer cells cannot be distinguished from the non-invasive ones, while spheroid condition amplifies the difference in terms of migration pattern, MMP secretion, mRNA profiling and immunostaining. Actin cytoskeleton machinery is found to be indispensable while acto-myosin and MMP mediated pathways are not critically important and can be by-passed easily by the invasive spheroids. Profile of mRNA of focal adhesion proteins changed significantly from 2D to 3D condition and might need more probing. Distinct focal adhesion complex is not observed in spheroid and their role needs to be reviewed cautiously. We conclude that 3D spheroid has the capacity to distinguish between invasive and non-invasive population and can be used as a cellular migration model.

## Electronic supplementary material

supplementary Info
