# Supplementary material for: Inherent aggressive character of invasive and non-invasive cells dictates the in vitro migration pattern of multicellular spheroid
Source: Sci Rep. 2017 Sep 14;7:11527. doi: 10.1038/s41598-017-10078-7 (PMC5599661; doi:10.1038/s41598-017-10078-7)
Supplement: Supplementary file 1 — supplementary Info [file 41598_2017_10078_MOESM1_ESM.pdf]

<u>Supplementary information</u>

Inherent aggressive character of invasive and non-invasive cells dictates the in vitro migration pattern of multicellular spheroid

Sukanya Gayan, Abhishek Teli, Tuli Dey*

Institute of Bioinformatics and Biotechnology, Savitribai Phule Pune University

SI Figure 1

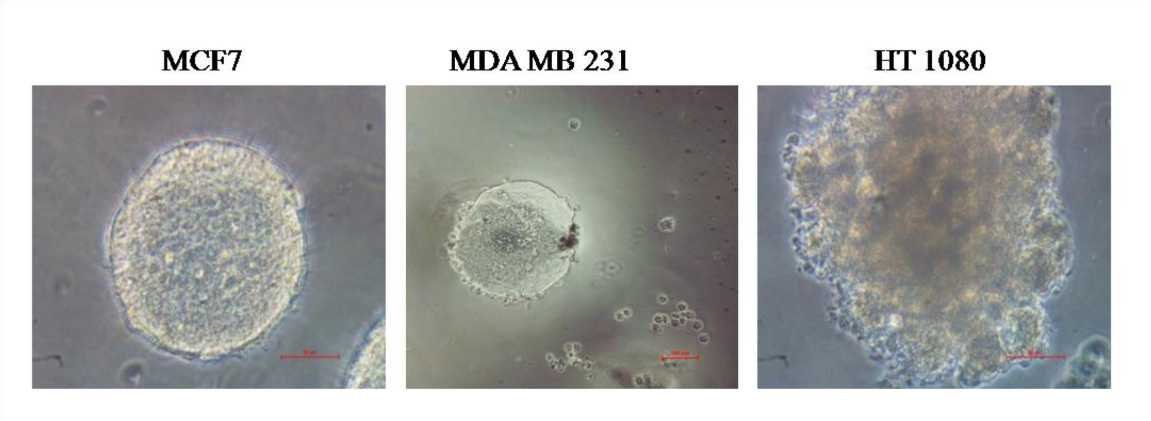

Fig 1 Phase contrast microscopic images of MCF7, MDA MB 231 and HT 1080 cells in 2D culture and as spheroid in agar coated plate.

SI Figure 2

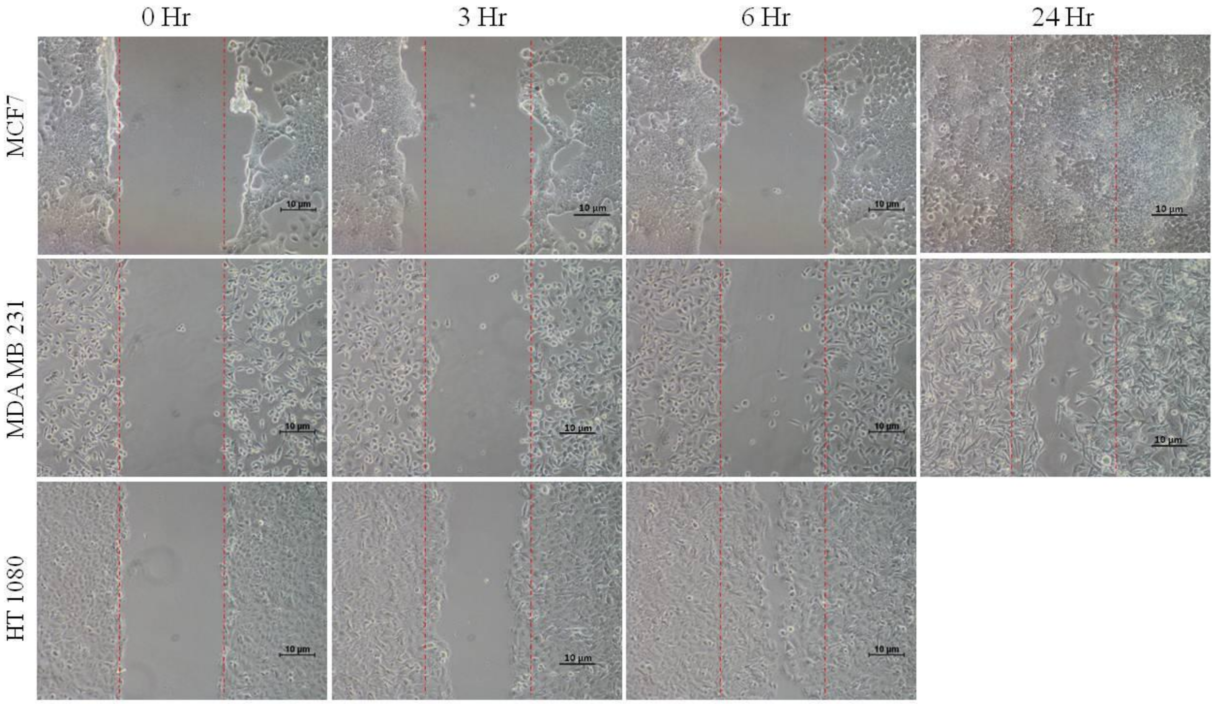

Fig 2 Cellular migrations analysis of MCF7, MDA MB 231 and HT 1080 cells in 2D platform. Confluent cultures are subjected to wound formation and subsequent healing over 0-24 hr. Imaging is done with phase contrast microscope (Nikon Eclipse TU1). Scale bar is 10 µm.

SI Figure 3

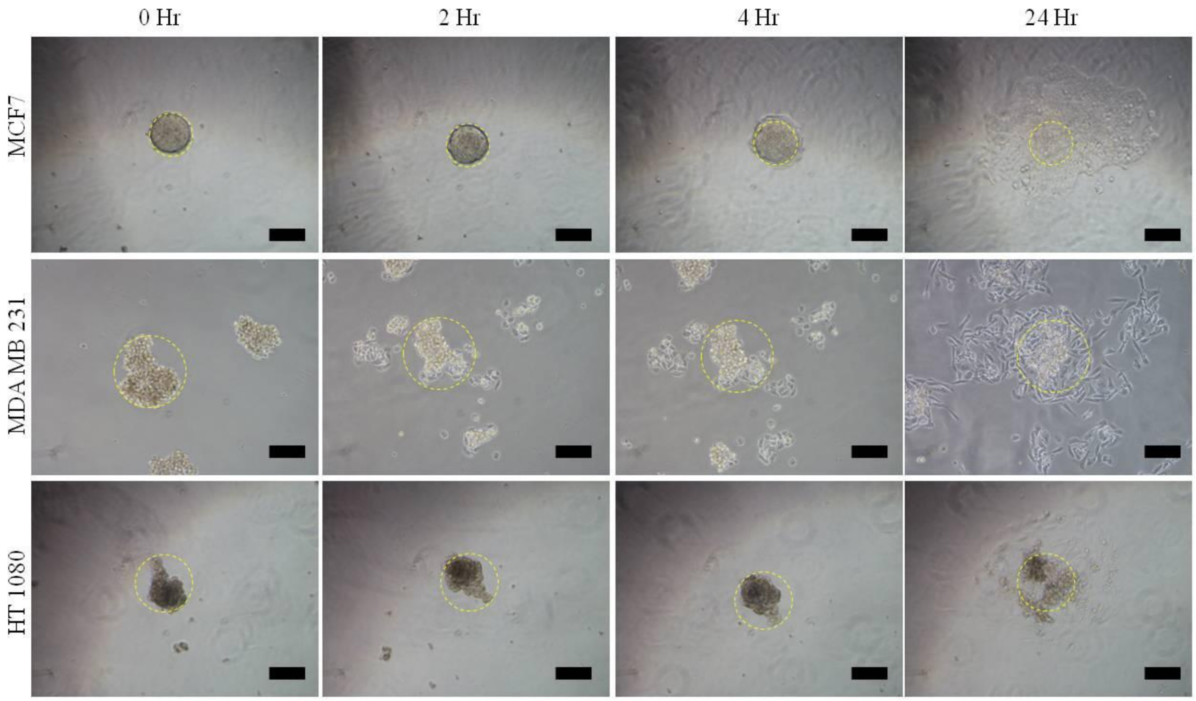

Fig 3. Pseudo 3D migration of MCF7, MDA MB 231 and HT 1080 spheroid on 2D surface. (A) Single spheroid is incubated on glass cover slip and incubated for 24 hr under standard cell culture condition. Disassociation/melting of spheroids are followed by coverage of surrounding area. Imaging of identified spheroids is done over time with phase contrast microscope (Nikon Eclipse). Scale bar is 10 µm.

SI Figure 4

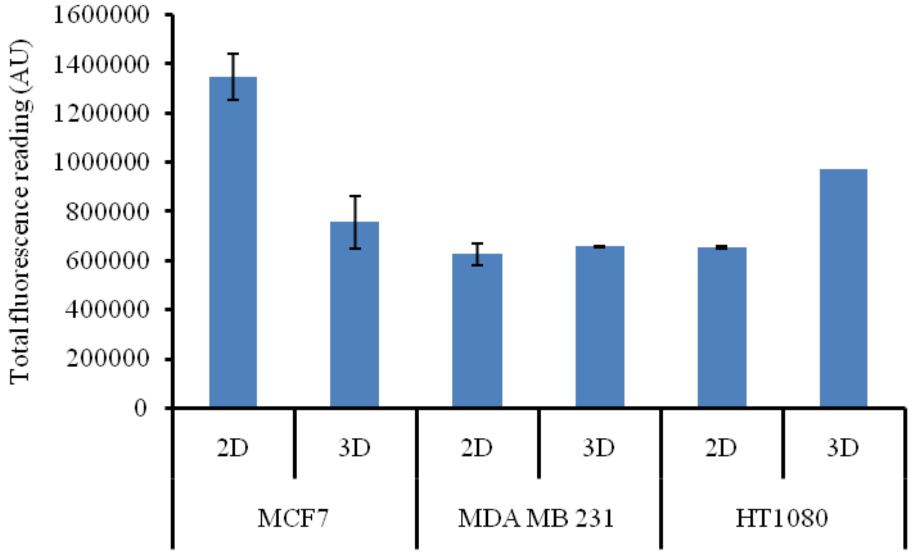

Fig 4. Comparative analysis of total fluorescence measurement of vinculin signal in MCF7, MDA MB 231 and HT 1080 cell, in 2D and 3D condition. Confocal image (green laser) was converted to 16 bit grey scale image in ImageJ. The mean grey intensity was then quantified and plotted.
